# Systematic tailoring for the implementation of guideline recommendations for anxiety and depressive disorders in general practice: perceived usefulness of tailored interventions

**DOI:** 10.1186/1471-2296-14-94

**Published:** 2013-07-04

**Authors:** Henny Sinnema, Berend Terluin, Michel Wensing, Daniëlle Volker, Gerdien Franx, Anton van Balkom, Jacomine de Lange

**Affiliations:** 1 Netherlands Institute of Mental Health and Addiction Trimbos Institute, Trimbos, The Netherlands; 2 The EMGO Institute for health and care research (EMGO+); Department of General Practice, VU University Medical Centre, EMGO, The Netherlands; 3 IQ Healthcare, Radboud University, Radboud, The Netherlands; 4 The EMGO Institute for health and care research (EMGO+); Department of Psychiatry, VU University Medical Centre, EMGO, The Netherlands

**Keywords:** Anxiety disorders, Depressive disorders, Primary care, Implementation, Tailored interventions, Guidelines, Qualitative research

## Abstract

**Background:**

The uptake of guideline recommendations in general practice can potentially be improved by designing implementation interventions that are tailored to prospectively identify barriers. However, there is insufficient evidence regarding the most effective and efficient approaches to tailoring. Our study provides an insight into the usefulness of tailored interventions to prospectively identified barriers affecting the uptake of guideline recommendations for anxiety and depressive disorders experienced by general practitioners (GPs) in their local context.

**Methods:**

A qualitative study was conducted, in which 23 GPs gave informed consent and 14 finally participated. To explore the barriers affecting the uptake of guideline recommendations, a face-to-face interview was conducted with each GP to generate a personalised list. In response to this list, interventions were tailored to remove the barriers experienced by the GPs. To examine the perceived usefulness of the tailored interventions, telephone interviews were conducted after one year and coded through thematic coding. The analysis was descriptive in nature.

**Results:**

The most frequently perceived barriers were: a lack of knowledge and skills, no agreement on guideline recommendations, negative outcome expectancy, low self-efficacy, no consensus with patients, and a lack of information about treatments provided by mental health professionals, together with waiting lists. The tailored interventions ‘peer group supervision’ and ‘individualised telephone consultations’ were perceived as useful by most GPs. Besides the tailored interventions, a perceived benefit of using a self-rating scale, measuring depressive and anxiety symptoms, and the idea of delivering better patient care, were supportive in the uptake of guideline recommendations.

**Conclusions:**

Our findings suggest that tailoring interventions to prospectively identified barriers, affecting the uptake of guideline recommendations for anxiety and depressive disorders, as perceived by GPs, may enhance the implementation of these guideline recommendations.

## Background

In different countries, clinical practice guidelines for anxiety and depressive disorders are available in primary and secondary care [1-5]. These guidelines provide recommendations on the recognition, diagnosis and the treatment of anxiety and depressive disorders. The management of anxiety and depressive disorders by General Practitioners (GPs) is not always consistent with prevailing guidelines. A variety of factors can affect the uptake of guideline recommendations. These factors are related to four levels: the patient level, the professional (GP) level, the organisational level and the social level [6-13]. Patients do not always recognize themselves that they have a psychological problem and present mainly somatic symptoms. In addition, despite their psychological symptoms or diagnosis, patients may not perceive need for care. With respect to the level of the GPs, barriers may be for example, struggle to distinguish between ‘normal’ distress and depression requiring treatment and no agreement on making a diagnosis. With respect to the organisation level identified barriers may be insufficient collaboration with mental health professionals, and waiting lists for specialty mental health care. Finally, with respect to the social level barriers may be for example limited financial incentives. In addition, some recommendations in the guidelines have less support from research evidence, or may be perceived as being less attractive. It is important to overcome these factors, because enhancing guideline adherence yields to superior outcomes, is cost-effective, and leads to the reduction of the burden of the disease and to improved social functioning [14-16]. Several theories and models are available which explain the factors that may influence the implementation of change in health care: namely, those related to individual professionals, the social environment and the healthcare system. Most theories propose that implementation interventions are most effective, if they address the most important barriers for improvement in the targeted setting [17].

At the start of our study, we hypothesised that the uptake of guideline recommendations, and consequently the improvement of patient outcomes, can be achieved by designing implementation interventions that are tailored to identifiable barriers affecting the uptake of guideline recommendations in the local context of GPs [17,18]. Several randomised controlled studies have investigated the impact of tailored interventions to improve the quality of care [18]. Because the tailoring methods used in these studies are heterogeneous, there is insufficient evidence of the most effective and efficient approaches to tailoring, including how barriers should be identified and how interventions should be selected to address these barriers [17].

In the present qualitative study, we provide an insight into the local barriers associated with the uptake of guideline recommendations for anxiety and depressive disorders experienced by GPs, and the usefulness of tailored interventions to these prospectively identified barriers. We focused on the guideline recommendations for the recognition and diagnosis of the disorders, for stepped care treatment allocation, and for the provision of information to patients. The research questions were: i) What were the barriers affecting the uptake of guideline recommendations for patients with anxiety and depressive disorders as perceived by GPs? and, ii), What was the perceived usefulness of the offered tailored interventions, peer group supervision and telephone consultation, to implement the guideline recommendations?

## Methods

### Study design

The study was conducted parallel to a cluster randomised controlled trial (RCT) of tailored interventions, to improve the management of anxiety and depressive disorders in primary care (NTR1912) [19]. For the selection of participants, we refer to the study protocol. Cluster randomisation was applied at the level of the general practice organisation. A total of 23 general practices were included; 11 practices (23 GPs) were allocated to the control group and 12 practices (23 GPs) to the intervention group. All GPs received an educational intervention; including one day of training, written information and a flowchart for the recognition and treatment of anxiety and depressive disorders, according to the stepped care approach, at the start, and with feedback at six months. Only GPs who were randomised to the intervention group participated in the present study. All GPs gave informed consent, after anonymity and confidentiality was assured. A qualitative evaluation design was used to explore the perceived barriers associated with the uptake of guideline recommendations, and the perceived usefulness of the tailored interventions offered in response to the barriers.

### Clinical guidelines

The focus was on the uptake of the following four key guideline recommendations for anxiety and depressive disorders:

1. *The recognition of high-risk patients for anxiety or depressive disorders with the Four-Dimensional Symptom Questionnaire (4DSQ).* The principal aim of the 4DSQ is to distinguish between stress-related syndromes (denoted as ‘stress’, ‘burnout’, ‘nervous breakdown’) and psychiatric disorders (i.e. depression and anxiety disorders) [20]. The 4DSQ is a self-rating questionnaire measuring the four dimensions of common psychopathology: distress, depression, anxiety and somatisation. The Anxiety Scale contains 12 items and has a range of 0–24; a score ≥ 9 on the anxiety subscale is an indication of a probable anxiety disorder diagnosis. The Depression Scale contains 6 items and has a range of 0–12; a score ≥ 6 on the depression subscale is an indication of a probable depressive disorder diagnosis.

2. *To diagnose an anxiety disorder or a depressive disorder in the case scores on the 4DSQ subscales indicate this.* An appropriate diagnosis included an assessment of the severity of the disorder, registered as simple or complex in the case of an anxiety disorder, and mild or severe in the case of a depressive disorder. The severity of the disorder is assessed on the basis of the number and the nature of symptoms, general functioning, the course of the illness, the risk of relapse and comorbidity.

3. *Stepped care allocation.* Based on the severity of the disorder, treatment was to be offered according to a stepped care algorithm; starting with the least intensive treatment that was still expected to generate effects. Patients with a simple or mild disorder were to be offered interventions of low intensity. More intensive treatment options would be appropriate for patients who do not successfully respond to low-intensity interventions and for patients with a complex or severe disorder.

4. *Proper psycho-education on anxiety and depressive disorders.* GPs had to provide information to patients on diagnosis and stepped care treatment options for anxiety and depressive disorders.

### The tailoring process

The tailored intervention consisted of two parts. First, barriers associated with the uptake of guideline recommendations experienced by the GPs were identified during face-to-face interviews. For this purpose, an interview guide was developed by four researchers (DV, MW, GF, and HS). The interview was based on previously published barriers associated with the uptake of guideline recommendations for depressive and anxiety related disorders [8,10,21,22]. The interview guide contained the following categories of barriers: i) an attitude regarding the guideline recommendations; ii) knowledge and skills; iii) time constraints; iv) the patients opinion and behaviour as perceived by the GPs, and v) the collaboration with mental health professionals. Beside, one open-ended question was included to gain insight in other barriers.

The interviews were held at the baseline of the RCT and yielded a barrier list for each GP. The second part of the tailored intervention was aimed at overcoming the identified barriers, during the implementation process of the guideline recommendations. The tailored interventions consisted of ‘peer group supervision’ and ‘individualised telephone consultation’ tailored to the local barriers. Peer group supervision was provided by a GP on two instances and focused on the barriers as experienced concerning knowledge and skills, and the opinion and the behaviour of patients as perceived by the GPs. The content of the telephone consultation was developed by the researchers (BT, MW, AB, GF, and HS) and provided by the interviewers, every two months, for 15 minutes, throughout one year (from June 2010 until June 2011). In the phone call, the interviewers mapped the local implementation processes, fed this information back to the researchers, and offered advice to the GPs in return during the follow-up call. Whenever solutions to barriers did not appear to be successful, new solutions were developed and discussed with the GP during the next contact. With this ‘continuous’ feedback loop between the researchers, the interviewers, and the GPs, we tried to optimise the tailoring process.

### The interview team

The interviews were carried out by three female interviewers, two with a nursing background and one with a psychology background, and working as non medical quality improvement professionals in general practice. In the Netherlands it is common that non medical professionals support the GPs in general practice to improve the quality of care. Each interviewer was assigned to four general practices and had two principle tasks: to interview the GP and feed back the advice provided by the research team to the GP. Prior to the study, the interviewers were trained by the research team to execute the aforementioned tasks. First, to obtain insight in the experienced barriers associated with the uptake of guideline recommendations the interviewers were trained by conducting one interview each under life-supervision of BT with a GP who did not participate in the study. Second, to feed back the tailored interventions we had two meetings with the interviewers (one with AB and HS and one with BT and HS) to discuss how tailored interventions should be fed back. Third, to optimize the telephone interviews, to obtain insight in the usefulness of the tailored interventions, two meetings were held with the interviewers (one with JL and HS and one with JL). The interviewers and researchers discussed the findings and the interviewers received feedback to conduct the next interviews. In the first meeting the interviewers indicated difficulty in performing two tasks. GPs were open in how they experienced the tailored interventions and particularly the negative comments felt as a condemnation, because the interviewers had offered the interventions and support. Accordingly, in the next interviews the interviewer interviewed the GPs which she had not offered tailored interventions. As a consequence, the interviewers felt more open to ask questions to experiences.

### Data collection

Data collection consisted of in-depth face-to-face interviews and in-depth telephone interviews with GPs. The face-to-face interviews were conducted in the general practice, prior to the tailoring of interventions, to get an insight into the barriers associated with the uptake of guideline recommendations (from May 2010 till August 2010). The interviews lasted for about 45 minutes, were digitally recorded, and then summarised in writing by the interviewers. To check the quality of the reports, three recorded interviews, from three different interviewers, were transcribed verbatim, and compared with the written reports by the principal investigator (HS). The reports appeared to be a good and satisfactory reflection of the conversation; therefore, the other digitally recorded interviews were not transcribed verbatim.

In-depth telephone interviews, to get insights of the perceived usefulness of the offered tailored interventions, followed a year after the delivery of the tailored interventions (from May 2011 till August 2011). For these telephone interviews, a topic list was developed for each GP (BT, HS) that consisted of questions related to barriers indicated previously by the GP, to the tailored interventions offered in response to the barriers, to the factors that might have influenced the implementation process, and finally, to the impact of the changes on patient care as perceived by the GPs. All of the telephone interviews lasted 30 minutes and were digitally recorded and transcribed verbatim.

### Data analysis

Data from the face to face interviews was categorised, following the barriers listed in the interview guide: attitude; knowledge and skills; time constraints; patient’s opinion and behaviour, and collaboration with mental health professionals. From these categories, sub-categories were derived. For example, ‘a lack of agreement about the diagnosis’ and ‘a lack of self-efficacy’, were gained as the sub-categories from ‘attitude’. New themes mentioned by the GPs such as ‘availability of treatment’ were added to our analysis. Others, such as ‘financial structures’ were not, since tailoring interventions to financial barriers was beyond the scope of our study. A mid-term analysis was conducted after four and after eight interviews, to generate an in-depth understanding that could be used in future interviews.

Data from the telephone interviews was coded through thematic coding, using the qualitative data analysis software programme MAXQDA 2007 [23]. The interview transcriptions were coded independently by two raters (HS and JL) and reflective notes were written to gain distance from the data. Constant comparative techniques were used to ascribe text extracts to the themes [24]. After the first eight interviews, similarities and differences between the researchers’ interpretations were discussed. Based upon agreements, a code-tree was developed and used for the analysis of the remaining interviews. The main categories in the code-tree were ‘supportive’ and ‘not supportive’ in the uptake of guideline recommendations. Text was coded to the category ‘supportive’ when the GPs mentioned that the offered tailored interventions were supportive, and when factors positively influenced the change process. New categories, derived from the data, were ‘the perceived benefit of using the 4DSQ’ and ‘the idea of delivering better patient care’. The analysis was descriptive in nature.

## Results

### Participant characteristics

The sample consisted of 23 GPs, 8 women and 15 men. The mean age for women was 45 years with a standard deviation of 10 (range 36 – 59 years). The mean age for men was 52 years with a standard deviation of 9 (range 29 – 63 years). Six GPs had solo practices and the others worked with other GPs in the same practice, with a maximum of eight GPs participating in one practice. Eight of 12 were rural practices, 4 practices were located in urban areas. From the 23 GPs, 19 were interviewed to establish the barriers. Four GPs did not participate, because 3 GPs from one practice had doubts about their participation in the study, and one GP had a lack of time (colleagues represented her). After one year, of the 19 included GPs, 14 GPs (three women), were interviewed to get an insight into the perceived usefulness of the tailored interventions. Five GPs were not interviewed; one was represented by a colleague due to limited time (they indicated having similar experiences with the tailored interventions), two had insufficient time, one became ill and one migrated.

## Barriers affecting the uptake of guideline recommendations before tailoring

Different barriers were perceived by the GPs to the uptake of guideline recommendations. GPs (n = 19) indicated a total of 84 barriers. The mean number was 4.4 barriers with a median of 4 barriers. Most GPs indicated barriers in i) using the 4DSQ (n = 15), ii) diagnosing anxiety and depressive disorders (n = 13) and iii) allocating patients correctly to care, according to the severity of the disorder diagnosed (n = 15). Only some GPs perceived barriers in providing patient information (n = 5). The various barriers were classified according to the themes: knowledge and skills, attitude, time, patient’s opinion and behaviour, collaboration with mental health professionals, and the availability of treatment.

### Lack of knowledge and skills

Most of the GPs (n = 16) perceived barriers related to a lack of knowledge and skills: in using the 4DSQ and in the interpretation of its scores (n = 8), to diagnose depression and anxiety, and as a part of the diagnostic process, to determine the complexity of anxiety disorders and to determine the severity of depression (n = 10), and in stepped care allocation to treatments (n = 7). Others indicated barriers such as an insufficient knowledge of: patient information (n = 3), e-mental health (n = 2) and how to motivate patients to change their behaviour (n = 4).

### Attitudinal barriers toward using the 4DSQ, diagnosing, providing psycho-education and allocating stepped care

Almost one third of the GPs (n = 9) experienced attitudinal barriers, such as a lack of agreement, of outcome expectancy and of self-efficacy. Two GPs did not use the 4DSQ. One, because he thought that ‘using the 4DSQ was not proper in patients who have experienced traumatic life events’, and the other GP mentioned that ‘he knew his patients and he hardly met patients with new psychiatric disorders’. A few GPs (n = 2) preferred not to diagnose patients, because they did not want to ‘label’ them with a ‘disorder’. For this same reason, written psycho-educational brochures about depression or anxiety disorders were not handed-out. One GP expressed a lack of self-efficacy in structuring the diagnostic, whereas two other GPs perceived stepped care allocation as standardised care, and rather provided care in accordance with the patient’s preference.

### Lack of time

Only a few GPs (n = 3) indicated barriers related to time. Two GPs indicated that informing patients with psychological complaints was time consuming and another had no time to provide brief interventions.

### Patient’s opinion and behaviour according to GPs

Almost one third of the GPs (n = 8) mentioned several barriers related to the patient’s opinion and behaviour. Some patients were not willing to complete the 4DSQ, or to schedule a next consultation to discuss the 4DSQ scores. Other patients disagreed with the diagnosis; especially those patients with a depressive disorder or unexplained physical complaints. Besides, some patients resisted prescribed treatment with an antidepressant.

### Collaboration with mental health professionals

Almost one third of the GPs (n = 11) perceived barriers related to collaboration with mental health professionals. First, the GPs missed information regarding which treatments mental health professionals in primary care provide. Second, the workload of the mental health nurse in primary care was too onerous or the GPs lacked a mental health nurse in their practice. Third, waiting lists for the primary care psychologist or specialised mental healthcare.

### Lack of availability of treatment

A few of the GPs (n = 3) indicated a lack of availability of brief interventions in primary care, and treatment opportunities for patients with severe mental health problems in the region.

## Usefulness of the tailored interventions

In general, the ‘peer group supervision’ and the ‘individualised telephone consultation’ were perceived as being useful for most of the GPs. The repeated calls worked out as useful reminders to perform according the guideline recommendations. In addition, the perceived benefit of using the 4DSQ, and the idea delivering better patient care, were supportive to the uptake of guideline recommendations. The perceived usefulness of the different tailored interventions and the factors that positively influenced the uptake of guideline recommendations are described below.

### Peer group supervision

Supervision was focused on the barriers related to knowledge, skills, patient’s opinion and behaviour. In total, more than half (n = 8) of the GPs participated in the peer group supervision. For all of the GPs, supervision was supportive in solving barriers in using the 4DSQ because the GPs: i) experienced time to focus on the 4DSQ; ii) heard from other GPs about how to handle the 4DSQ and iii) the GPs developed skills in how to interpret the 4DSQ scores. The GPs indicated that the interaction between colleagues in a small group was supportive.

*“The understanding of the 4DSQ scores gives a better picture of the anxiety, depression or somatisation complaints; the 4DSQ differentiates and gives direction to which treatment is indicated. Before the supervision, I often thought in terms of depressive complaints only”* (GP 9).

For all of the GPs, except one, supervision was also supportive in solving barriers in diagnosing mental disorders and stepped care allocation. The GPs acquired more insight into finding an agreement with the patient on the diagnosis, in diagnosing different types of anxiety disorders by asking practical basic questions, and in determining the complexity and severity of the disorders.

*“I have more knowledge of the different subtypes of anxiety disorders and, as a consequence, I have identified more patients with an anxiety disorder and provided treatment. And, because I have more knowledge myself, I can explain the anxiety disorder to the patient with more confidence”* (GP 11).

*“Now, I am better aware of the complexity of anxiety disorders and what kind of treatment is necessary”* (GP 3).

One GP was less positive about the supervision, having expected more attention being paid to the principles of stepped care treatment. He experienced that the training, provided before the start of the project to all participating GPs, was more helpful in this respect, just like the flowchart for recognising, diagnosing and stepped care treatment allocation for anxiety and depressive disorders.

Less than half of the GPs (n = 6) did not participate in the supervision offered as part of the tailoring process, mostly for practical reasons such as lack of time. These GPs perceived this lack of supervision as a barrier for gaining sufficient knowledge about working with the 4DSQ. They somewhat compensated for this by seeking advice from colleagues, from the mental health nurse working in their practices, and by telephone consultation.

### Telephone consultation

All of the GPs received personalised telephone consultations to remove the implementation barriers perceived by them. More than half of the GPs (n = 9) indicated that the consultation was supportive, and most of them (n = 6) found that the calls worked out as useful reminders. For less than half of the GPs (n = 5), the consultations were not supportive.

*“The telephone contact had an added value in addition to the supervision. I was not always happy with the reminders, but they ensured that the issue remained under my attention, and made clear what I still had to figure out and that is important for the implementation. The use of the 4DSQ has become a part of my work, because I was called back every time”* (GP 2).

### Consultation related to a lack of knowledge and a lack of time

The GPs who perceived barriers in diagnosis, stepped care allocation and having insufficient knowledge of brief interventions, were reminded to use the previously offered flowchart for recognising, diagnosing, and stepped care treatment allocation for anxiety and depressive disorders. Most of the GPs indicated that the flowchart was helpful.

*“The flowcharts lie on my desk and they provide support in determining the severity of the condition and the appropriate treatment step to allocate, especially when I see a patient with an anxiety or a depressive disorder”* (GP 12).

Some of the GPs indicated that the flowchart added a little.

*“Stepped care is in my head. Occasionally, I look afterwards to the flowchart to check if I forgot something”* (GP10).

Besides the use of the flowchart, the GPs who indicated having insufficient knowledge of brief interventions and a lack of time were also given advice about the treatment interventions that they could provide: for example, psychoeducation, including written or online information, watchful waiting, e-health interventions and a referral to social work. One GP perceived the advice on psycho-education as being only a little supportive, because most patients were not self-sufficient individuals. Most of the GPs experienced that this helped them to improve their care. Their provision of information and their referral opportunities were improved and they did not prescribe antidepressants when these were not indicated.

“*I developed a brochure for myself with information about anxiety and depression, adding also the psycho-education for patients*” (GP3).

*“Because I gained a better insight into the diagnosis and associated treatment options, I now succeed more often to motivate patients for treatment. Also, I am more relaxed towards patients, because I have more knowledge of the different treatment options, including watchful waiting. I used to refer quickly or gave an antidepressant”* (GP 10)*.*

The GPs were also informed about the possibility of following a course in problem solving therapy at the Dutch College of General Practitioners. However, none of the GPs followed such a course, due to a lack of time.

### Consultation related to attitudinal barriers

The telephone consultations were also helpful in addressing some of the attitudinal barriers experienced by the GPs. Explanation on the use of the 4DSQ, the need to diagnose, to allocate stepped care, and to inform patients about the diagnosis and treatment options were supportive.

*“I am less hesitant to label patients with a diagnosis. Both the 4DSQ, the flowchart and subsequently the dialogue with the patient help me to come to an agreement about the diagnosis and the severity of the depressive disorder and about the treatment options”* (GP 4).

One GP though, was convinced that patients give socially desirable answers on rating scales and, therefore, he did not trust the 4DSQ and only relied on his own clinical assessment.

*“I am faithful to my own intuition. I look to the scores, but listen more to my inner voice. The 4DSQ apparently provides a grip where I am not looking for. I listen to what I find”* (GP 14).

### Consultation related to patient’s opinion and behaviour

Advice about how to solve barriers in using the 4DSQ consisted of asking patients why they did not want to fill out the 4DSQ; respect for their refusal and the use of intervention watchful waiting; and to make clear which patients should be approached actively to discuss the 4DSQ score and to make an appointment for a follow-up consultation. The advice was supportive, albeit not always in patients with a complex personality.

*“This woman is always difficult to motivate for things other than those she has in mind. In my opinion, she is a difficult woman. Most patients accept to fill out the 4DSQ”* (GP 4).

The GPs who mentioned barriers in finding agreement about the diagnosis were advised to give patients an active role by referring patients to written or online psycho-education and different treatment options; and to ‘act as an advisor’ so that the patient could make his/her own decisions. The GPs indicated that these pieces of advice were less supportive in solving the barriers.

### Consultation related to collaboration with mental health professionals

The GPs who had insufficient information about who can do what in primary care, or who had to deal with a waiting list, or those who missed a mental health nurse in general practice, were given the advice to make a social map and to organise a meeting with mental health professionals in primary care to discuss who can provide what. Some of the GPs, or their mental healthcare nurse (n = 4), made a social map of mental health professionals to whom they could be referred, to create more treatment opportunities.

*“I made a list of psychologists in this neighbourhood and had a meeting with social workers. Thereafter, I refer more to social workers”* (GP 5).

The GPs indicated that the opportunity to refer to a mental health nurse or psychologist in general practice facilitates stepped care allocation. Because the professional is located in the practice, consultation is easy to organise. Besides, the mental health professionals can provide care quickly, the lines are short.

*“A mental health nurse has come in my practice and by discussing patients, my knowledge has increased about what I can do and what others can do”* (GP 7).

### Consultation related to a lack of availability of treatment

The GPs who had a lack of availability of interventions received information on which other interventions could be provided and to which other professionals they could be referred. The advice was supportive, but so also was the reduction of the waiting list for specialised mental health care.

#### Benefit of using the 4DSQ

Almost all GPs experienced the 4DSQ as a useful tool (n = 13). They indicated that the 4DSQ structured the dialogue with the patient. The 4DSQ scores gave the GP and the patient information about present complaints, and the severity, which was useful when coming to an agreement about whether or not psychological complaints were part of patient’s problem. The scores gave a direction to the diagnosis and treatment. The GPs indicated that they had something to offer patients and that patients felt understood.

*“A patient with a chronic obstructive pulmonary disease, who was a frequently consulter, experienced I as a whiner. After administering the 4DSQ, I discovered he was anxious. When I discussed this with the patient, he finally felt understood. With the 4DSQ, I get a grip on the problem and the feeling that I have something to offer the patient”* (GP 2).

#### Delivering better care

Most of the GPs (n = 10) indicated that, in their opinion, patients received better care because the GPs had more insight into diagnosis and associated treatment options. Therefore their motivation to diagnose increased.

*“I diagnose more often, so I can explain why which treatment options are indicated. I inform patients more than before participation in the project”* (GP 8).

## Discussion

### Summary of main findings

The present research involved a qualitative evaluation of GPs’ experience with tailoring interventions, to prospectively identified barriers, affecting the uptake of four key guideline recommendations for anxiety and depressive disorders. Barriers were mostly related to knowledge and skills, attitude, patient’s opinion and behaviour according to the GPs, and the collaboration with mental health professionals. Based upon the barriers, peer group supervision and individualised telephone consultations, tailored to the experienced barriers of each GP, were chosen as implementation strategies to improve the uptake of the guideline recommendations. For most of the GPs, peer group supervision was supportive in teaching them how to use the 4DSQ in their consultations with patients, how to diagnose different types of anxiety disorders, to determine the complexity and severity of the disorders, and how to share experiences with other GPs. More than half of the GPs indicated that individualised telephone consultations were supportive, and worked out as useful reminders and as incentives, to change fixed patterns. The perceived benefit of using the 4DSQ, and the idea of delivering better care, were essential factors to overcome the identified barriers and were supportive in the uptake of guideline recommendations.

### Strengths and limitations

There is a lack of knowledge about effective ways to identify implementation barriers and how to select interventions likely to overcome them [18]. A strength of this study is the use of a systematic tailored approach to this problem, consisting of: i) identification of the barriers affecting the uptake of guideline recommendations, ii) designing implementation interventions appropriate for these barriers, and iii) the application and evaluation of implementation interventions that are tailored to the identified barriers. The qualitative approach, incorporating two interview moments, provided a deeper understanding of the barriers, and solutions affecting GPs uptake of guideline recommendations, before and during the implementation process. Another strength is that all of the GPs were interviewed, thus providing the maximum insight into the perceived usefulness of the tailored interventions. The participating GPs were representative for all Dutch GPs regarding age. However, the percentage of GPs with a solo practice was somewhat higher than average (26% versus 18%), and respondents may have differed regarding other not measured variables. GP and practice characteristics may have influenced the results. The study has a number of limitations. First, all of the GPs were more or less motivated to implement guideline recommendations by participating in the study, which hampers the study’s generalisation to other GPs. Second, to provide an insight into the perceived barriers, we focused on a limited number of barriers derived from the literature. Obviously, more factors influence the uptake of guideline recommendations, such as financial structures, but these are more difficult to modify in the context of a clinical study. Finally, there was no validation by case record review to corroborate participants reports.

### Comparison with existing literature

The perceived benefit of using the 4DSQ and the idea delivering better care appeared to be essential factors associated with the uptake of guideline recommendations. It is widely known that a relative advantage is an important factor in the implementation of innovations, though a relative advantage alone does not guarantee widespread adoption [25]. In our study, it seemed that the GPs were interested in using the guideline recommendations, especially the 4DSQ, and during their use, the GPs developed a positive attitude. With the 4DSQ GPs received a tool to distinguish between stress-related syndromes and psychiatric disorders. Distinguish between ‘normal’ distress and depression requiring treatment was one of the barriers in recognising and diagnosing depression, Barley et. al. (2011) found in their meta-synthesis of research to identify barriers and facilitators [12]. Our finding that most GPs were interested, developed a positive attitude towards guideline recommendations and indicated that their performance had changed, is in line with the ten-stage model for planning change. This model is a synthesis of different stages of change models, which offer theoretical assumptions about the steps professionals must take to implement the intended changes [26]. To maintain the achieved change, the ten-stage model suggests a reminder system. In our study, the telephone consultations were perceived as useful reminders and were supportive in changing the fixed patterns that the GPs indicated.

Different theories regarding a change in healthcare, such as the stages-of-change theories, can be used in planning and evaluating changes in clinical practice. Overall, there seem to be two approaches for tailoring in implementation science. The first approach, used by some implementation scientists, suggests a wider use of theory in implementation research; the second approach, used by others, represents a pragmatic and empirical approach to implementation science. Evidence to support any or either approach is limited [17]. In our study, we adopted the second approach; studying the uptake of guideline recommendations pragmatically, by building on already known factors, instead of linking to theoretical perspectives.

An important assumption, underlying tailoring, is that implementation interventions are most helpful if these effectively address the most important determinants of practice for improvement in the targeted setting [18]. Although, most of the GPs in our study perceived that the tailored interventions of supervision and telephone consultation as being supportive, it does not mean that these interventions effectively addressed all of the prospectively identified barriers affecting the uptake of guideline recommendations. In a multiple case analysis, Bosch et al. (2007) suggested that there is often a mismatch between identified barriers for change and the implementation interventions chosen [27]. Nevertheless, as the barriers largely differ within guidelines, we applied a tailored and barrier-driven implementation strategy, focusing on perceived barriers in daily practice, and adapting the strategy according to perceived success [28].

### Implications for future research

In our study, we used a qualitative research method for systematic tailoring. The systematic qualitative procedure is known to be more time consuming than surveys. Future research could be focused on the development of validated questionnaires for the uptake of mental health guideline recommendations in primary care.

## Conclusion

Tailoring supervision and telephone consultations to personally perceived implementation barriers may be supportive in the uptake of guideline recommendations by GPs. The perceived benefit of using the 4DSQ and the idea of delivering better care to patients appeared to be essential factors associated with the uptake of guideline recommendations.

### Ethical principles

The qualitative study is a part of the RCT which has been approved by the Medical Ethical Committee of the Institutions for Mental Health, in 2009 (METiGG; Utrecht, the Netherlands).

## Competing interests

BT is the copyright owner of the 4DSQ and receives copyright fees from companies that use the 4DSQ on a commercial basis (the 4DSQ is freely available for non-commercial use in health care and research). BT received fees from various institutions for workshops on the application of the 4DSQ in primary care settings.

## Authors’ contributions

HS contributed to the design of the study, participated in the training of the interviewers, contributed to the development of the tailored interventions, performed the analysis and wrote this article. BT contributed to the design of the study, participated in the training of the interviewers, contributed to the development of the tailored interventions and co-authored the article. MW contributed to the design of the study, participated in the development of the tailored interventions and co-authored the article. DV contributed to the design of the study and co-authored the article. GF contributed to the design of the study, participated in the development of the tailored interventions and co-authored the article. AvB contributed to the design of the study, participated in the training of the interviewers, contributed to the development of the tailored interventions and co-authored the article. JL contributed to the design of the study, participated in the training of the interviewers, contributed to the development of the tailored interventions, performed the analysis and co-authored the article. All authors read and approved the final manuscript.

## Pre-publication history

The pre-publication history for this paper can be accessed here:

http://www.biomedcentral.com/1471-2296/14/94/prepub
